# Late Ca^2+^ Sparks and Ripples During the Systolic Ca^2+^ Transient in Heart Muscle Cells

**DOI:** 10.1161/CIRCRESAHA.117.312257

**Published:** 2018-02-01

**Authors:** Ewan D. Fowler, Cherrie H.T. Kong, Jules C. Hancox, Mark B. Cannell

**Affiliations:** From the School of Physiology, Pharmacology & Neuroscience, Faculty of Biomedical Sciences, University of Bristol, University Walk, United Kingdom.

**Keywords:** action potential, cardiac myocytes, heart, heart muscle cell, sodium–calcium exchanger

## Abstract

**Rationale:**

The development of a refractory period for Ca^2+^ spark initiation after Ca^2+^ release in cardiac myocytes should inhibit further Ca^2+^ release during the action potential plateau. However, Ca^2+^ release sites that did not initially activate or which have prematurely recovered from refractoriness might release Ca^2+^ later during the action potential and alter the cell-wide Ca^2+^ transient.

**Objective:**

To investigate the possibility of late Ca^2+^ spark (LCS) activity in intact isolated cardiac myocytes using fast confocal line scanning with improved confocality and signal to noise.

**Methods and Results:**

We recorded Ca^2+^ transients from cardiac ventricular myocytes isolated from rabbit hearts. Action potentials were produced by electric stimulation, and rapid solution changes were used to modify the L-type Ca^2+^ current. After the upstroke of the Ca^2+^ transient, LCSs were detected which had increased amplitude compared with diastolic Ca^2+^ sparks. LCS are triggered by both L-type Ca^2+^ channel activity during the action potential plateau, as well as by the increase of cytosolic Ca^2+^ associated with the Ca^2+^ transient itself. Importantly, a mismatch between sarcoplasmic reticulum load and L-type Ca^2+^ trigger can increase the number of LCS. The likelihood of triggering an LCS also depends on recovery from refractoriness that appears after prior activation. Consequences of LCS include a reduced rate of decline of the Ca^2+^ transient and, if frequent, formation of microscopic propagating Ca^2+^ release events (Ca^2+^ ripples). Ca^2+^ ripples resemble Ca^2+^ waves in terms of local propagation velocity but spread for only a short distance because of limited regeneration.

**Conclusions:**

These new types of Ca^2+^ signaling behavior extend our understanding of Ca^2+^-mediated signaling. LCS may provide an arrhythmogenic substrate by slowing the Ca^2+^ transient decline, as well as by amplifying maintained Ca^2+^ current effects on intracellular Ca^2+^ and consequently Na^+^/Ca^2+^ exchange current.

## Abstract 2

Supplemental Digital Content is available in the text.

Cardiac excitation–contraction coupling is mediated at the cellular level by the near-synchronous activation of ≈10^4^ microscopic Ca^2+^ release events called Ca^2+^ sparks.^1,2^ This occurs because the cardiac action potential (AP) opens L-type Ca^2+^ channels (LTCC) in the surface membrane to produce a local increase in Ca^2+^, which in turn opens Ca^2+^-sensitive channels (ryanodine receptors) in the adjacent junctional sarcoplasmic reticulum membrane (jSR).^3^ The spatial restrictions associated with this “local control mechanism” provide this signal transduction pathway both high gain and stability^1,4^ and forms the cornerstone of our current understanding of excitation–contraction coupling, explaining the time- and voltage-dependence of the regenerative Ca^2+^ release process.^5–7^ Ca^2+^ sparks normally occur with high probability at the start of the Ca^2+^ transient,^8,9^ and Ca^2+^ release during the Ca^2+^ spark is terminated in ≈10 ms, probably via SR depletion–dependent processes.^10^ The cytoplasmic Ca^2+^ concentration then returns toward resting levels in a few hundred milliseconds as Ca^2+^ is pumped back into the SR (via SERCA2a [the sarco/endoplasmic reticulum Ca ATPase]) and across the surface membrane (mainly via NCX [sodium/calcium exchanger]).^4,11,12^ This currently accepted view of excitation–contraction coupling has led to changes in the time course of Ca^2+^ decline being generally attributed to changes in SERCA2a and NCX activities with smaller contributions from a sarcolemmal Ca^2+^-ATPase and mitochondria.^13,14^ However, continued SR release (or leak) should oppose SR reuptake and slow the time course of the Ca^2+^ transient, as seen in a phospholamban knockout mice with CamKIIδc overexpression.^15^

**Meet the First Author, see p 386**

## Methods

The data that support the findings of this study are available from the corresponding author on reasonable request.

Ventricular cardiomyocytes from New Zealand White rabbits were field-stimulated at 0.5 Hz at 1.2× threshold at 22°C. The enzymatic method for isolating rabbit epicardial myocytes have been described previously.^16^ To avoid possible problems associated with cell dialysis, whole-cell patch clamp techniques were not used. Cell movement artifacts were prevented by adding 10 mmol/L 2,3-butanedione monoxime to normal Tyrode superfusion solution. It should be noted that late Ca^2+^ sparks (LCSs) were also seen in the absence of 2,3-butanedione monoxime and at 37°C (see Online Figure I). 2,3-Butanedione monoxime may modify the relative importance of triggers for Ca^2+^ sparks in the experiments shown here (see Online Supplement). Fast block of LTCCs was achieved by local superfusion with Cd^2+^ (10 or 100 μmol/L) plus 1 μmol/L sulforhodamine-B in normal Tyrode superfusion solution from a pressurized micropipette, the fluorescence of which allowed determination of the local concentration of Cd^2+^ (and hence degree of LTCC block).

More extensive Methods are given in the Online Supplement.

## Results

**Figure 1.**
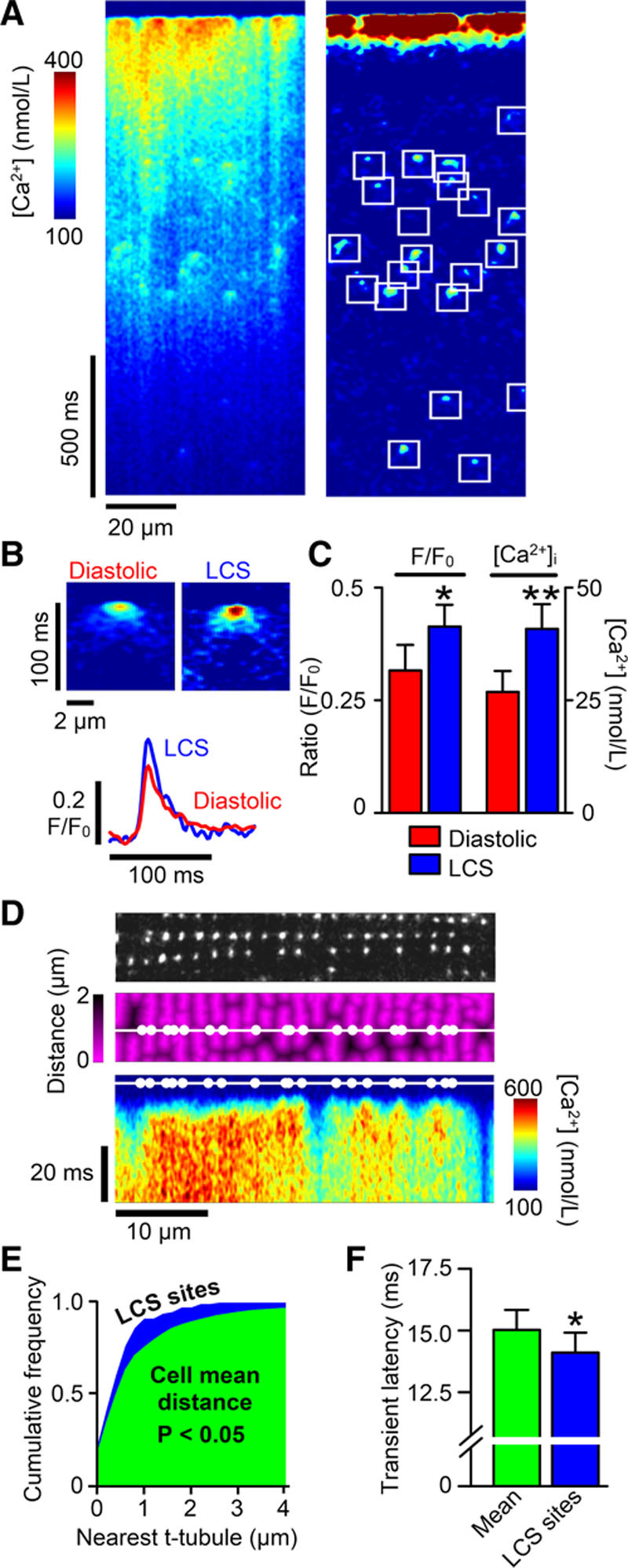
**Late Ca^2+^ sparks (LCS) during the decay of evoked Ca^2+^ transients.** **A**, High-resolution recordings (confocal pinhole set to 1 Airy unit) of Ca^2+^ release during normal Ca^2+^ transients (**left**) at 0.5 Hz. Image enhancement by subtraction of the low-pass filtered transient shows LCS more clearly (white boxes; **right**). **B**, Comparison of Ca^2+^ sparks at rest (**top left**) and LCS (**top right**) showed high spatiotemporal similarity. The temporal profiles of averaged events (23 Ca^2+^ sparks and 18 LCS) show that LCSs have a similar time course to resting Ca^2+^ sparks in the same cell (**bottom**). **C**, Average LCS amplitude was greater than resting Ca^2+^ sparks (*P<0.05, **P<0.01, 196 LCS, 42 diastolic n/N=12/6). **D**, Di-8-ANEPPS (4-(2-[6-(dioctylamino)-2-naphthalenyl]ethenyl)-1-(3-sulfopropyl)pyridinium) labeling of t-tubules (**top**) was used to calculate a distance map to nearest t-tubule (**middle**). The line scan position is indicated by the white line and the location of LCS by white circles. LCS occurred near t-tubules and at regions with early evoked Ca^2+^ release (**bottom**). **E**, The median distance to nearest t-tubule was shorter for LCS sites than the cell average distance (n/N=11/5). Kolmogorov–Smirnov test. **F**, Ca^2+^ transient latency at sites with LCS was shorter than the cell average latency (n/N=28/8). *P<0.05.

By using high sensitivity detectors and improved confocality, we have been able to detect additional Ca^2+^ release events (LCSs) during the entire time course of the cellular Ca^2+^ transient. The left panel in Figure 1A shows a typical Ca^2+^ transient recorded from a ventricular myocyte using confocal line scanning,^9^ and the right panel shows a high-pass filtered and contrast-enhanced version of this image in which these LCS can be seen more clearly. Individual LCS (Figure 1B) had an increased amplitude compared with diastolic Ca^2+^ sparks (Figure 1C) but did not exhibit any changes in duration (≈40 ms) or spatial full width at half maximum (≈1.8 μm; data not shown). The increase in amplitude of the LCS can be explained by a reduction in cytoplasmic Ca^2+^ buffering power because of cytoplasmic Ca^2+^ binding sites (such as troponin) becoming occupied during the Ca^2+^ transient.^14^ A simple, obvious, explanation for the genesis of the LCSs would be that they arise from jSR that was either not activated during the upstroke of the Ca^2+^ transient or was uncoupled or orphaned^17^ from t-tubules (which carry the AP to the cell interior). To examine this idea, we labeled t-tubules (Figure 1D) and measured the Euclidian distance to the LCS centroid (Figure 1E) and compared the latencies for the upstroke of the Ca^2+^ transient at LCS sites to overall Ca^2+^ transient latency (Figure 1F). Perhaps unexpectedly, LCS occurred close to t-tubules and at positions where the Ca^2+^ transient developed with a shorter than average latency (*P*<0.05). This suggests that LCS usually arise from sites that are not orphaned but are, in fact, well coupled, a view also supported by the presence of an extensive t-tubule network (top and middle panels, Figure 1D) that did not show any signs of the disruption associated with orphaning.^17^

**Figure 2.**
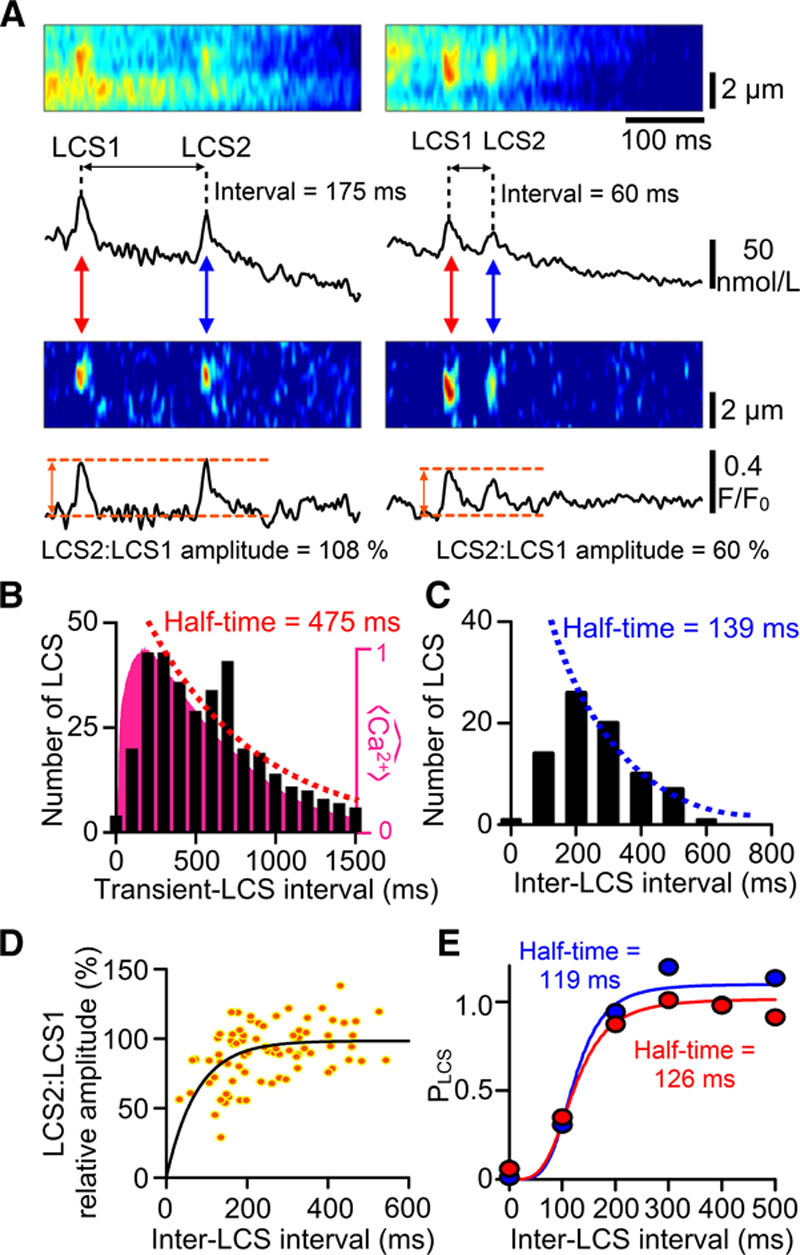
**Restitution and refractory behavior of late Ca^2+^ sparks (LCS).** Time and amplitude restitution of LCS reveal their sarcoplasmic reticulum (SR) load dependence. **A**, The interval of successive LCS arising from the same location (**top**), and their relative amplitudes were measured in background-subtracted recordings (**bottom**). **B**, The probability density function of LCS after evoked Ca^2+^ release was similar to the time course of the normalized average Ca^2+^ transient (solid color behind histogram bars) and decreased exponentially from the maxima (red line). **C**, The probability of a second LCS at the same site had a similar time dependence to the first LCS (**B**). **D**, After the first LCS, the amplitude of the second LCS increased with a time constant, τ=77 ms. **E**, The probability of LCS activation (*P*_LCS_) was essentially the same after evoked Ca^2+^ release (red) or after a prior LCS (blue; *P*=0.86, extra sum-of-squares *F* test). **B**, 350 LCS from n/N=19/9; **C** and **D**, 79 LCS pairs from n/N=10/8.

After activation, Ca^2+^ spark sites enter a refractory period,^18,19^ which should oppose any secondary activation. The spontaneous Ca^2+^ spark refractory period has been estimated by analyzing the inter-Ca^2+^ spark interval from ryanodine-modified rat^18^ or CamKIIδc-overexpressing mouse^15^ myocytes. We performed a similar analysis on LCS sites: Figure 2A shows 2 LCS sites with different inter-LCS intervals. Initial LCS (red arrows) followed the AP-evoked Ca^2+^ transient with a latency distribution shown as in Figure 2B. Only a few LCS are seen shortly after the onset of the Ca^2+^ transient, but this increases and reaches a peak before declining with a half time of ≈500 ms, which is similar to the half time of the whole-cell Ca^2+^ transient (also shown in Figure 2B behind the histogram bars) in this species at room temperature.^20^ The time between the first and second LCS (blue arrows Figure 2A) is shown in Figure 2C, and the amplitude of the second event compared with the first is shown in Figure 2D. Because LCS amplitude restitution matches the reported rabbit jSR refilling time course,^19^ jSR refilling probably determines LCS amplitude recovery. In this regard, LCS behave similarly to ryanodine-stimulated diastolic Ca^2+^ sparks.^18^ By dividing Ca^2+^ spark probability by an exponential fit, the first and second LCS activation probability can be derived.^18^ It is apparent that the LCS activation probability is (essentially) the same for both the first and second LCS (Figure 2E), suggesting that recovery from refractoriness is a dominant factor in their genesis—but what is their trigger?

**Figure 3.**
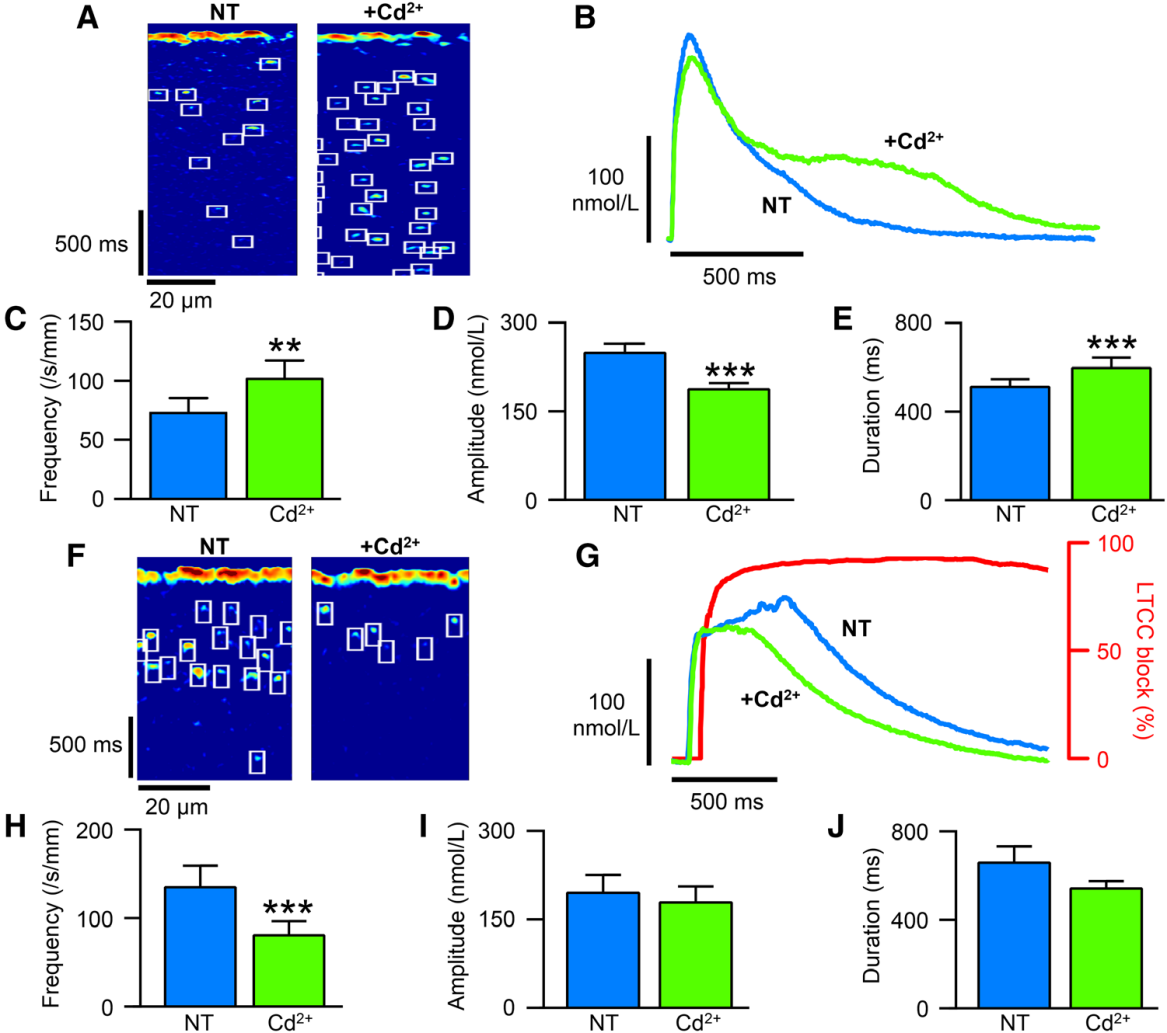
**Effects of L-type Ca^2+^ channel (LTCC) block reveals additional triggers for late Ca^2+^ sparks (LCS).** Blocking LTCC before or after evoked Ca^2+^ release shows LCS can be triggered by late LTCC activity. **A**, Line scan recordings of consecutive Ca^2+^ transients in normal Tyrode’s (NT) and in NT+10 μmol/L Cd^2+^. The extracellular solution was rapidly changed between contractions to preserve sarcoplasmic reticulum (SR) load. Cd^2+^ application produced more spatial nonuniformities in early Ca^2+^ release and increased the number of LCS. **B**, The Ca^2+^ transient decay was clearly delayed in Cd^2+^. **C**, Cd^2+^ increased mean LCS frequency by ≈40%. **D**, Cd^2+^ slightly decreased the Ca^2+^ transient amplitude and (**E**) increased its duration compared with NT. **F**, Rapid application of NT+100 μmol/L Cd^2+^ (after the Ca^2+^ transient upstroke) preserved early Ca^2+^ release but decreased the number of LCS. **G**, The Ca^2+^ transient upstroke was preserved in Cd^2+^ (green line) compared with NT (blue). **H**, Mean LCS frequency was reduced by ≈40% by Cd^2+^. **I**, Average Ca^2+^ transient amplitude was not reduced by rapid Cd^2+^ application while the Ca^2+^ transient duration was decreased slightly (**J**; *P*=0.05). **C**–**E**, n/N=15/5; **H**–**J**, n/N=12/4. **P<0.01, ***P<0.001 paired *t* test.

During the long plateau phase of the AP, LTCCs continue to open stochastically^21,22^ and might trigger LCS as individual release sites recover from their refractory period. If this is the case, one might predict that LTCC inhibition should reduce the number of LCS. As illustrated in Figure 3A, blocking ≈75% of LTCCs with extracellular Cd^2+^ (a fast LTCC open-state blocker^23^) paradoxically increased the number of LCS (Figure 3C). However, straightforward interpretation of this experiment is complicated by the block of LTCCs during the upstroke of the AP, reducing jSR site activation (Figure 3D). This effect would increase release site availability because fewer sites would then be refractory at later times. Importantly, these data also show that the increased number of LCS produced in this condition make a significant contribution to the time course of the Ca^2+^ transient (Figure 3B and 3E). To remove the complication arising from changes in the number of jSR release sites activated during the AP, we rapidly applied a higher concentration of Cd^2+^ just after the upstroke of the Ca^2+^ transient to selectively block later LTCC openings. To show the effect of such early LTCC blockade clearly, we show an exemplar (Figure 3F) that had a higher rate of LCS production shortly after the upstroke of the Ca^2+^ transient, which contributed to an extended rising phase which can be seen under condition of lower SR load (eg, Grantham and Cannell^21^). The time of arrival and local concentration of Cd^2+^ was measured by fluorescently labeling the solution with sulforhodamine-B which, with the *K*_d_ for Ca^2+^ block, allowed us to estimate that ≈90% LTCCs should be blocked after the upstroke of the Ca^2+^ transient (Figure 3G). This reduced the number of LCS, but only by 40% (Figure 3H), supporting the idea that LCS can also be triggered by the rise in cytosolic Ca^2+^ during the Ca^2+^ transient. As expected, the application of Cd^2+^ shortly after the stimulus and upstroke of the AP had no effect on the amplitude of the Ca^2+^ transient (Figure 3I) but slightly shortened its duration (Figure 3G and 3J), which is likely to be as result of AP shortening^24^ (because of LTCC blockade) and the reduced number of LCS.

**Figure 4.**
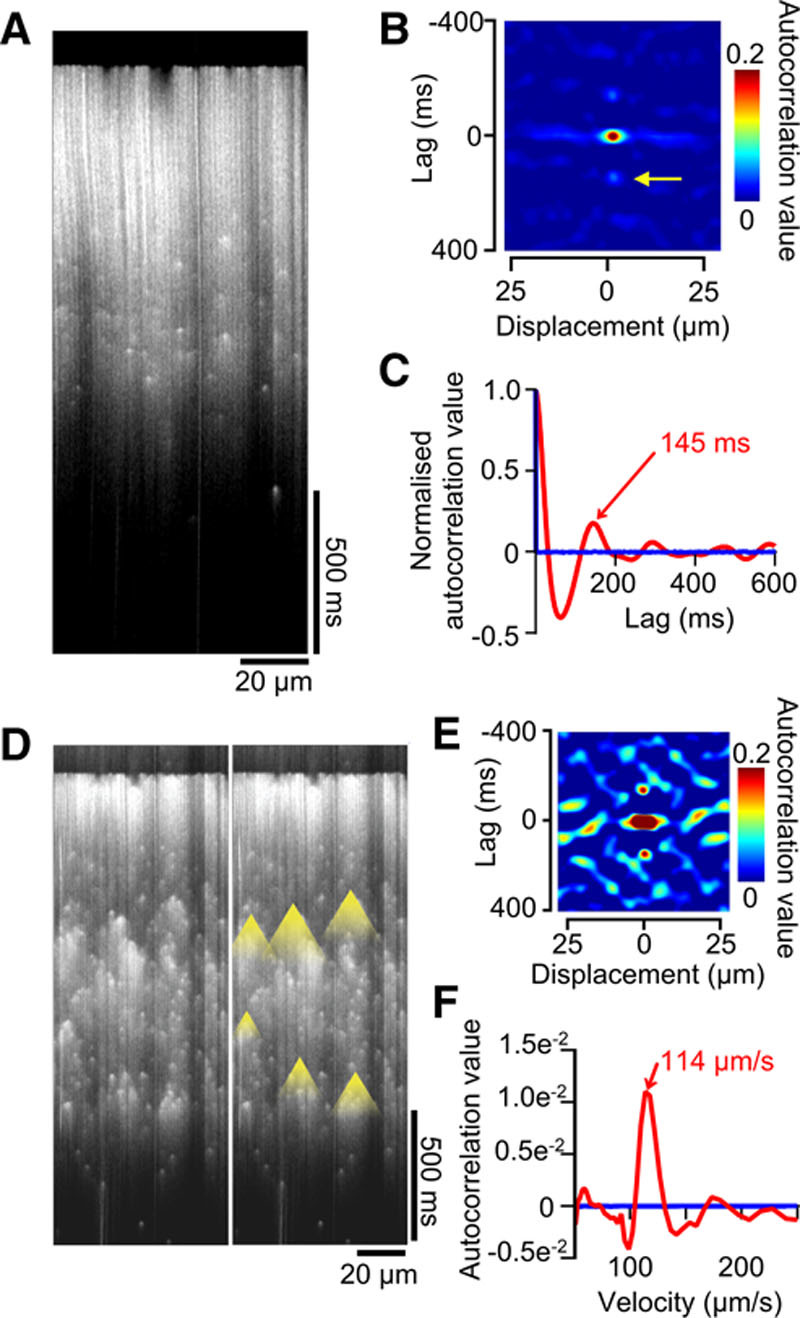
**Late Ca^2+^ sparks (LCSs) can interact to form Ca^2+^ ripples.** **A**, Unprocessed high resolution recording showing multiple LCS. **B**, The autocorrelogram of (**A**) reveals the time dependence between repeating events (indicated by yellow arrow), with a mean delay of 145 ms, as highlighted in (**C**). **D**, **left**, Further pacing resulted in increased Ca^2+^ load and many more LCS. **Right**, Emphasizes how LCS appear to trigger additional sites forming multiple propagating Ca^2+^ ripples (marked by yellow chevron overlays). **E**, The 2D autocorrelogram shows that some LCS have both temporal and spatial relationships to other LCS. **F**, Calculation of propagation velocities in the 2D autocorrelation showed a dominant peak at ≈114 μm/s. Points indicated in (**C**) and (**F**) were highly significant (*P*<10^–5^) (blue lines show mean±5 SD of scrambled data).

LCS production is a part of a continuum of behavior that spans the low spontaneous Ca^2+^ spark rate during diastole (≈1 per 100 μm/s scanned) to high rates (≈10^4^ per 100 μm/s) during the upstroke of the Ca^2+^ transient.^8,9^ Normally, Ca^2+^ sparks interact weakly,^25^ but when SR and cytoplasmic Ca^2+^ levels increase, Ca^2+^ waves can develop from the sequential recruitment of Ca^2+^ spark sites.^26^ The spatiotemporal relationship between LCS site activation (Figure 4A) was analyzed by calculating autocorrelograms (Figure 4B). In most of our experiments, such autocorrelation analysis showed only a time-dependent relationship between spark sites, reflecting the refractory period (Figure 4C) described earlier. However, in a subset of cells that were more highly Ca^2+^ loaded (Figure 4D), the autocorrelogram showed multiple peaks, indicating that some LCS were both spatially and temporally correlated (Figure 4E). The right panel of Figure 4D illustrates the chevron patterns in LCS production that can be seen by eye, and analysis of the 2D autocorrelogram showed an apparent propagation velocity between LCS sites ≈114 μm/s (Figure 4F), similar to typical macroscopic Ca^2+^ wave propagation velocities.^26,27^ We call these novel propagating LCS events Ca^2+^ ripples as (1) they are smaller in amplitude, (2) do not propagate over the entire cell, and (3) occur during the declining phase of the Ca^2+^ transient, although they are clearly related to the well-known phenomenon of Ca^2+^ waves which can occur during the diastolic period in cardiac myocytes.^3,26,27^

## Discussion

The presence of LCS during the normal Ca^2+^ transient has important implications for understanding the complex interplay of SR Ca^2+^ release site activation, refractoriness, SR Ca^2+^ reuptake, and triggers for CICR (Ca-induced Ca release; see Online Figure II). Reduced LTCC activation cannot only disrupt the initial phase of Ca^2+^ release, leading to dyssynchrony,^28,29^ but also increase the number of LCS that can slow the decline of the Ca^2+^ transient. It is notable that reduced LTCC activation can also occur with pathological changes in t-tubules,^30^ APs,^13^ or signal transduction cascades.^3^ Increased SR leak in a CamKIIδc-overexpressing mouse model has been shown to slow the decline of the Ca^2+^ transient, and some LCS activity during the Ca^2+^ transient can be seen in Figure 7 of that paper.^15^ These results are also consistent with the ability of release sites to recover from refractoriness sufficiently quickly for some fraction to become reactivated either by cytosolic Ca^2+^ or LTCCs. While some uncertainty exists in the relative roles of cytoplasmic Ca^2+^, LTCC, and NCX in triggering LCS and their effect on the Ca^2+^ transient time course (an uncertainty compounded by 2,3-butanedione monoxime used to inhibit movement artifacts—see Online Supplement), it is clear that LCS production will be sensitive to all of these triggers. In the case of heart failure, any increase in LCS production could exacerbate the existing problem of slowed Ca^2+^ reuptake because of decreased SERCA2a activity.^31^ Further complexity is added by the changes in Ca^2+^ transient time course also affecting LTCC gating via prolongation of the AP because of NCX-generated current during the declining phase of the Ca^2+^ transient,^13^ as well as the differential responses of coupled and uncoupled release sites.^32^

Under normal conditions, LCS production is initially inhibited by the refractory period after Ca^2+^ spark activation,^18^ but the time course of recovery is shorter than the duration of the plateau of the AP during which a sizeable LTCC current flows. Thus, LCS are more likely to occur late in the AP, and slowing the decline of the Ca^2+^ transient may contribute to the antagonism between inward NCX current and repolarization reserve.^13^ As illustrated in Online Figure II, some pathological changes in the excitation–contraction coupling cycle could increase the probability of LCS which, in turn, may prolong the duration of the Ca^2+^ transient^15^ and AP duration. This forms a new positive feedback pathway that will promote AP prolongation and further Ca^2+^ influx via LTCC, further destabilizing Ca^2+^ cycling and increasing all forms of Ca^2+^ leak.^33^

While more work is needed to fully explore the implications of the novel results presented here, it is now apparent that SR Ca^2+^ release in the form of LCSs can continue at lower rates throughout the cardiac Ca^2+^ transient rather than solely during the upstroke of the Ca^2+^ transient as usually modeled.

## Acknowledgments

This work was supported by the British Heart Foundation (grant RG/12/10/29802) and Medical Research Council (MR/N002903/1).

## Disclosures

None.

## Supplementary Material

## Novelty and Significance

### What Is Known?

- Ca^2+^ transients are because of near-synchronous Ca^2+^ release from internal stores soon after the upstroke of the action potential.
- Ca^2+^ changes during the Ca^2+^ transient reflect the balance of release and uptake mechanisms, with the latter dominating the time course of the decline in Ca^2+^.
- Ca^2+^ dysregulation in cardiac muscle cells influences contraction strength and can promote fatal arrhythmias.
