# Supplementary material for: Late Ca2+ Sparks and Ripples During the Systolic Ca2+ Transient in Heart Muscle Cells
Source: Circ Res. 2018 Feb 1;122(3):473–8. doi: 10.1161/CIRCRESAHA.117.312257 (PMC5796647; doi:10.1161/CIRCRESAHA.117.312257)
Supplement: Supplementary file 1 [file res-122-473-s001.pdf]

SUPPLEMENTAL MATERIAL

Detailed Methods

Animals

All experiments were performed in accordance with the UK Home Office Animals (Scientific Procedures) Act 1986 and with approval by the University of Bristol ethics committee. Left ventricular epicardial myocytes were obtained from adult male New Zealand white rabbit (2-2.5 kg) hearts after full anaesthesia (50 mg/kg sodium pentobarbital i.v.) and euthanasia. Enzymatic dissociation was carried out using 1 mg/ml collagenase II (Worthington), 0.05 mg/ml protease (type XIV Sigma) and 0.1 mmol/L Ca^{2+}, as described previously (1).

Solution changes and local perfusion

Experiments were performed in a modified Tyrode's solution (NT) (containing, in mmol/L: 133 NaCl, 5 KCl, 1 NaH_2PO_4, 10 HEPES, 10 glucose, 1.8 $CaCl_2$, 1 $MgCl_2$, pH 7.4 with NaOH) at 22±1°C. Solution changes were performed by local superfusion of cells in the recording chamber. A pressurized micropipette and a custom-made valve system allowed rapid exchange (half time < 40 ms) of the cell bathing solution synchronised to electrical stimulation. The superfusion pipette contained NT solution supplemented with 10 or 100 µmol/L $CdCl_2$ (from a 10 mmol/L stock in water) and 1 µmol/L sulforhodamine B. The time course of solution exchange was monitored during Ca^{2+} line scan recordings by alternating the excitation wavelength between 488 and 543 nm for each line, while collecting emission at 492-600 and >600 nm.

The fractional block of LTCC by Cd^{2+} was calculated from the Hill equation with half-maximal inhibition occurring at 2.14 µmol/L and a Hill coefficient of 0.74 (2). The onset of LTCC block during solution exchange was calculated from the normalised change in the sulforhodamine B fluorescence signal. Note that Cd^{2+} is a weak blocker of NCX (by <1 % and 15 %, at 10 and 100 µmol/L respectively (2)) and TTX-sensitive Na^+ channels (by <1 % and 23 %, at 10 and 100 µmol/L respectively (3)). Cd^{2+} was chosen over organic pharmacological blockers because LTCC block by Cd^{2+} is rapid and not voltage or use-dependent.

Confocal Ca^{2+} line scan recording

Cells were loaded with 5 µmol/L Fluo-4-AM for 15 min, washed in NT and then allowed to rested for >10 min to allow time for de-esterification. Ca^{2+} sparks and transients were recorded in line scan mode using an inverted confocal microscope (LSM 880, Zeiss) with a 1.4 NA 63x oil immersion lens. Excitation light was provided by a 488 nm argon laser and fluorescence emission collected at 492-600 nm. Ca^{2+} line scans were recorded with the pinhole set to <2 Airy units, at a pixel size of 0.1-0.2 µm/pixel and with a scan speed of 1-2 ms per line. GaAsP photodetectors were used to increase the sensitivity of Ca^{2+} spark detection. Ca^{2+} line scans were recorded with the pinhole set to <2 Airy units, pixel size <0.2 µm/pixel and scan speed of 1-2 ms/line. The local concentration of Cd^{2+} was measured from the included sulforhodamine-B fluorescence which was excited at 543 nm and emission at >600 nm.

Fluorescence image processing

Non-cell background fluorescence from an area adjacent to the cell was subtracted from recordings. Variations in fluorescence due to dye loading was minimised by normalising fluorescence (F) to resting fluorescence during a 100 ms quiescent period immediately before stimulation (F_0). The F/F_0 recording was converted into units of $[Ca^{2+}]$ using the self-ratio method (4):

$$[Ca^{2+}]_i = \frac{KR}{(K/[Ca^{2+}]_{rest}) - R + 1}$$

Where *K* is the *in vivo* affinity of Fluo-4 for Ca^{2+} (K_d ~1000 nmol/L), *R* is the self-ratio fluorescence (F/F_0), and $[Ca^{2+}]_{rest}$ is the resting Ca^{2+} concentration (~100 nmol/L) (5).

During systolic Ca^{2+} transients, the increased cytosolic $[Ca^{2+}]$ presents a challenge for the detection of LCS due to the reduced contrast of fluorescent Ca^{2+} dyes at high cytosolic $[Ca^{2+}]$. To partially ameliorate this problem, the low-frequency time-averaged fluorescence in x*t* line scan recordings was subtracted from the F/F_0 recording. A low-pass quadratic Savitsky-Golay filter (window size ~301 ms) was applied along the *t* dimension, for every point in the *x* dimension. These filter values were found to effectively suppress background fluorescence variation due to the underlying Ca^{2+} transient, while preserving the morphology and enhancing detectability of LCS.

<u>Ca^{2+} spark detection</u>

Ca^{2+} sparks were detected using an automated optimal filter algorithm implemented in MATLAB (described in detail elsewhere (6)). Briefly, the algorithm cross-correlated the flattened line scan recording with a model Ca^{2+} spark and the location with the greatest correlation was identified. Following a test for significance, the centroid and amplitude of the underlying Ca^{2+} spark was then measured and recorded. The Ca^{2+} spark identification process was repeated until the significance of the maximum correlation fell below the threshold of significance. Ca^{2+} spark full-width at half maximum and full-duration at half maximum were measured in the flattened line scan recordings. Ca^{2+} spark amplitude was measured in the original F/F_0 or $[Ca^{2+}]$ line scan recordings at the location of detected Ca^{2+} sparks.

<u>Ca^{2+} transient analysis</u>

Ca^{2+} transient latency was defined as the time between electrical stimulation and when cytosolic $[Ca^{2+}]$ was >5 standard deviations above the resting Ca^{2+} signal. Ca^{2+} transient latency was measured at every point along the scan line. Ca^{2+} transient duration was measured as the full-duration at half maximal fluorescence (time from 50 % peak to 50 % decay of the Ca^{2+} transient).

<u>T-tubule imaging and processing</u>

The t-tubule system in the area surrounding the Ca^{2+} line scan recording was imaged by labelling the sarcolemma with di-8-ANEPPS from a stock 1 mmol/L solution (in DMSO) added directly to the cell recording chamber (final concentration 1 μmol/L) for 2-3 min. A stack of *xy* images above and below the recording focal plane was recorded using 488 nm excitation and emission collected at >600 nm.

3D stacks of t-tubule images were deconvolved using a model point spread function for the microscope objective which was derived from images of 100 nm fluorescent beads. A 3D t-tubule detection algorithm was implemented in MATLAB by the authors (who can be contacted for further details). The resulting data was then skeletonized in 3D in MATLAB (Skeleton 3D, version 1.12).

The origin of Ca^{2+} sparks in three dimensions (*xyz*) is uncertain in line scanning due to the spatial spread of Ca^{2+} sparks from sites just outside the scanned line. To minimize this problem, the t-tubule skeleton was collapsed to form a maximal intensity projection of the region ±2 μm above and below, and ±1 μm adjacent to the line scan region to capture the possible location of all closely coupled jSR release sites. The Euclidean distance from the apparent LCS centroid to nearest t-tubule was calculated from this maximal intensity projection.

<u>LCS propagation/interaction analysis using 2D autocorrelation</u>

2D autocorrelation was used to analyze the temporal and spatial relations between late Ca^{2+} sparks and Ca^{2+} ripples in Ca^{2+} line scan records. The autocorrelogram axes represent time (lag) and space (displacement), hence the distance and angle of structures relative to the origin of the autocorrelogram corresponds to the dominant frequency and velocity of LCS propagation in the form of Ca^{2+} ripples. The autocorrelation values in the time domain were measured from the origin and normalised to the autocorrelation value at the origin. The angle from the origin to the peaks of the autocorrelation correspond to propagation velocities of Ca^{2+} ripples (~50-250 μm/s).

To test whether the apparent peaks in the autocorrelogram were significant, we scrambled the data and repeated the autocorrelation. This was repeated 5 times to compute a mean and standard deviation for equivalent uncorrelated data.

Statistical Analysis

Data were tested for normality using the Shapiro-Wilk test and in cases where data were skewed, the test was reapplied to log-transformed data. Paired t-tests were performed on normally distributed original data or the transformed data. Differences between cumulative frequencies were tested using the Kolmogorov-Smirnov test. Curve fits of LCS probability (P_{LCS}) were compared using the extra sum-of-squares F test. Results are presented as mean ± SEM. The number of cells (n) and animals (N) used for each experiment are given in figure legends as n/N. $p<0.05$ was considered to be the limit of statistical confidence. A nested hierarchical approach was used to examine possible clustering effects in Fig. 1 and the ICC was found to be only 0.14 (7).

Supplemental Text

Effects of BDM.

BDM is not a specific inhibitor of muscle contraction (8) and partially inhibits many of the key Ca^{2+} transport systems in cardiomyocytes. It has been shown to affect NCX (9), Ca^{2+} channels (10) and weakly enhances PP1 and/or PP2A phosphatase activity (the latter at 100 mmol/l) (11). A reduction in LTCC availability should increase the probability of LCS as shown in Fig. 3a. On the other hand, 10 mM BDM can reduce SR Ca^{2+} content with only small effects on the amplitude of the Ca^{2+} transient (12). Therefore, BDM may alter the exact relationship between triggers and the consequent Ca^{2+} release but is clearly not the cause of LCS activity seen here (see Online Figure I).

Supplemental Figures and Figure Legends

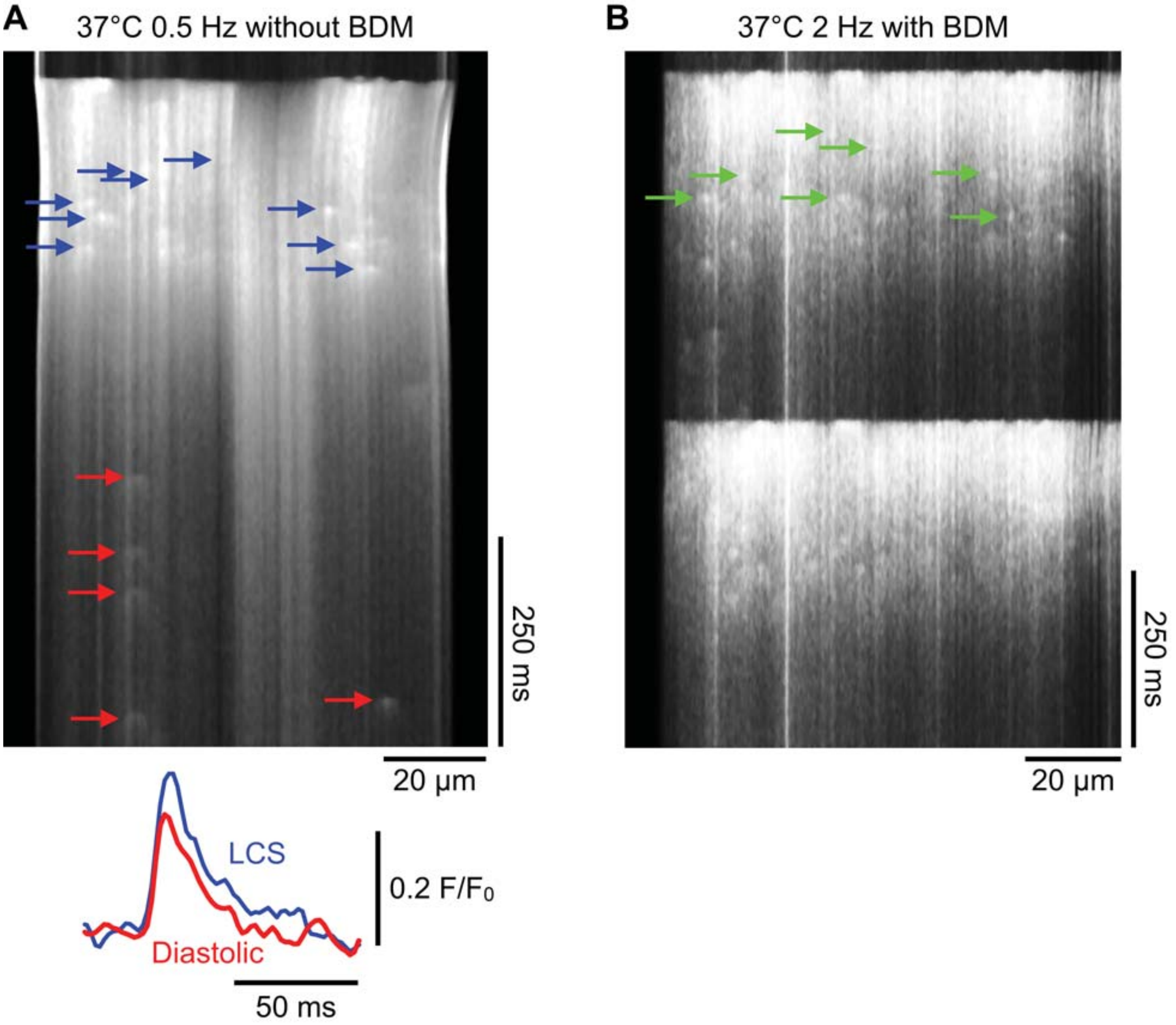

Online Figure I . Examples of LCS production at 37°C. The left hand panel shows unprocessed data in the absence of BDM with some clear LCS indicated by blue arrows. Note the presence of a movement artifact showing the contraction of the cell. For comparison, some diastolic Ca^{2+} sparks are also indicated (red arrows). Plots of average amplitudes show similar behavior to the data shown in Figure 1. On the right, the pacing rate was increased to 2Hz at 37°C (with BDM to avoid the movement artifact) and, while the increased Ca^{2+} transient amplitude decreases contrast and increases Poisson noise, LCS can still be discerned.

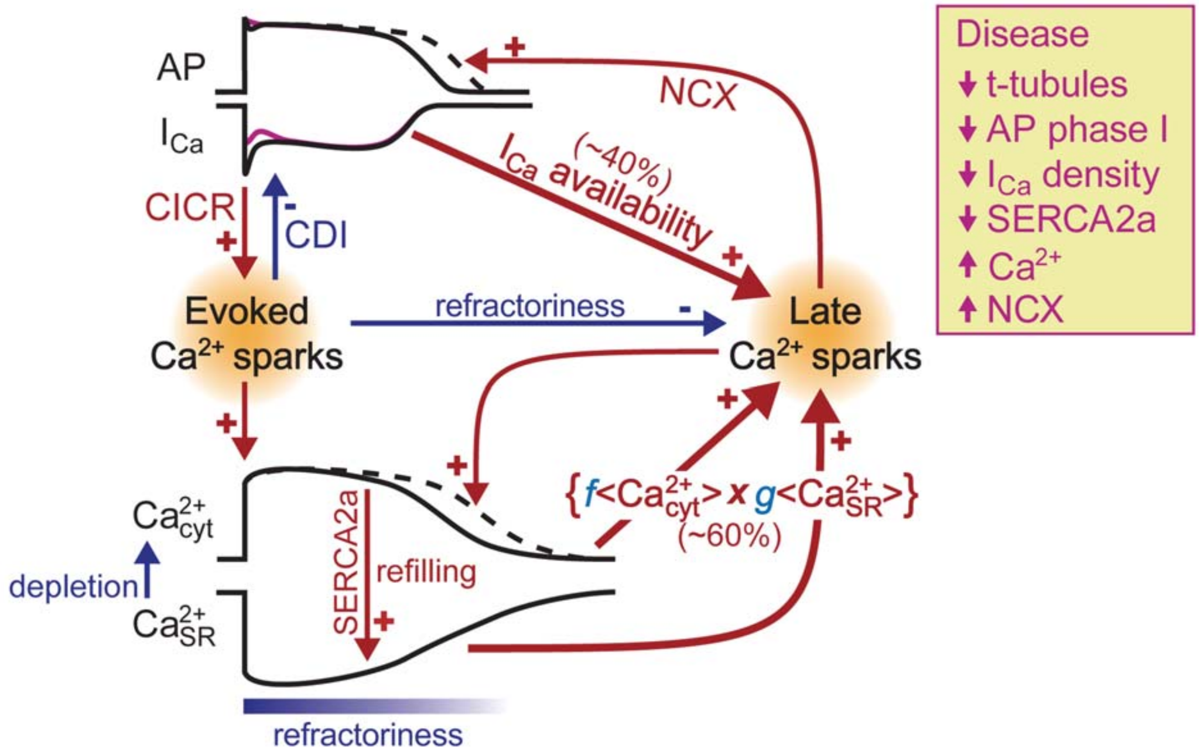

Online Figure II. Mechanisms for interactions between Ca^{2+} sparks, LCS and the ECC cycle. During the cardiac AP, Ca^{2+} enters the cell via LTCC (seen macroscopically as I_{Ca}) which causes the near-synchronous release of Ca^{2+} from jSR by Ca^{2+} -induced Ca^{2+} release (CICR). Activated SR junctions enter a refractory state where further release is prevented due to depletion of SR Ca^{2+}. Ca^{2+} -dependent inactivation (CDI) of LTCC may also decrease the probability of LCS. Cytosolic Ca^{2+} is re- sequestered by SERCA2a, refilling the SR. During the AP plateau, LTCC may (re-)open and may trigger LCS if the SR release sites were triggered during the AP or else recovered from the refractory state. Inward current generated by NCX will prolong the AP and increase the duration of I_{Ca}. By delaying the decline of the Ca^{2+} transient, LCS may promote additional LCS which may take the form of Ca^{2+} ripples, if SR load is sufficient and SR release not refractory. The non- LTCC triggered probability of LCS triggering can be described by an equation of the form $f<Ca^{2+}{}_{cyt}>$ x $g< Ca^{2+}{}_{SR}>$ where f and g describe the cytosolic and SR dependencies of Ca^{2+} spark initiation. This may account for ~60% of LCS in normal cells (the remainder being due to LTCC activity). In disease, the pathological changes in the ECC cycle can increase the probability of LCS which, in turn, may prolong the duration of the Ca^{2+} transient and AP duration forming a new positive feedback pathway.

Supplemental References

1. Cooper PJ, Soeller C, Cannell MB. Excitation-contraction coupling in human heart failure examined by action potential clamp in rat cardiac myocytes. *J Mol Cell Cardiol* 2010;49:911–917.

2. Hobai IA, Bates JA, Howarth FC, Levi AJ. Inhibition by external Cd2+ of Na/Ca exchange and L-type Ca channel in rabbit ventricular myocytes. *Am J Physiol: Heart Circ Physiol* 1997*;*272:H2164–H2172.

3. DiFrancesco D, Ferroni A, Visentin S, Zaza A. Cadmium-induced blockade of the cardiac fast Na channels in calf Purkinje fibres. *Proc Roy Soc Lond, B* 1985;223:475–484.

4. Cannell MB, Cheng H, Lederer WJ. Spatial non-uniformities in [Ca2+]i during excitation-contraction coupling in cardiac myocytes. *Biophys J* 1994;67:1942–1956.

5. Wier WG, Cannell MB, Berlin JR, Marbán E, Lederer WJ. Cellular and subcellular heterogeneity of [Ca2+]i in single heart cells revealed by fura-2. *Science* 1987;235:325–328.

6. Kong CHT, Soeller C, Cannell MB. Increasing Sensitivity of Ca2+ Spark Detection in Noisy Images by Application of a Matched-Filter Object Detection Algorithm. *Biophys J* 2008;95(12):6016–6024.

7. Sikkel MB, Francis DP, Howard J, Gordon F, Rowlands C, Peters NS, Lyon AR, Harding SE, MacLeod KT. Hierarchical statistical techniques are necessary to draw reliable conclusions from analysis of isolated cardiomyocyte studies. *Cardiovasc Res*. 2017;113:1743–1752. doi:10.1093/cvr/cvx151.

8. Sellin LC, McArdle JJ. Multiple effects of 2,3-butanedione monoxime. *Pharmacol Toxicol* 1994;74(6):305–313.

9. Watanabe Y, Iwamoto T, Matsuoka I, Ohkubo S, Ono T, Watano T, Shigekawa M, Kimura J. Inhibitory effect of 2,3-butanedione monoxime (BDM) on Na(+)/Ca(2+) exchange current in guinea-pig cardiac ventricular myocytes. *Br J Pharmacol*. 2001;132:1317–1325.

10. Chapman RA. The effect of oximes on the dihydropyridine-sensitive Ca current of isolated guinea-pig ventricular myocytes. *Pflugers Arch* 1993;422:325–331.

11. Zimmermann N, Boknik P, Gams E, Gsell S, Jones LR, Maas R, Neumann J, Scholz H. Mechanisms of the contractile effects of 2,3-butanedione-monoxime in the mammalian heart. *Naunyn-Schmied Arch Pharmacol*. 1996;354:431–436.

12. Adams W, Trafford AW, Eisner DA. 2,3-Butanedione monoxime (BDM) decreases sarcoplasmic reticulum Ca content by stimulating Ca release in isolated rat ventricular myocytes. *Pflugers Arch* 1998;436:776–781.
